# The sodium–proton exchangers sNHE and NHE1 control plasma membrane hyperpolarization in mouse sperm

**DOI:** 10.1016/j.jbc.2024.107932

**Published:** 2024-10-28

**Authors:** Analia G. Novero, Paulina Torres Rodríguez, José L. De la Vega Beltrán, Liza J. Schiavi-Ehrenhaus, Guillermina M. Luque, Micaela Carruba, Cintia Stival, Iñaki Gentile, Carla Ritagliati, Celia M. Santi, Takuya Nishigaki, Diego Krapf, Mariano G. Buffone, Alberto Darszon, Claudia L. Treviño, Dario Krapf

**Affiliations:** 1 Instituto de Biología Molecular y Celular de Rosario, CONICET-UNR, and Laboratorio de Medicina Reproductiva, Facultad de Ciencias Bioquímicas y Farmacéuticas, UNR, Rosario, Argentina; 2 Instituto de Biotecnología, UNAM, Cuernavaca, México; 3 Instituto de Biología y Medicina Experimental (IBYME-CONICET), Ciudad Autónoma de Buenos Aires, Argentina; 4 Department of Obstetrics and Gynecology, Washington University School of Medicine, St Louis, Missouri, USA; 5 Department of Electrical and Computer Engineering, Colorado State University, Fort Collins, Colorado, USA

**Keywords:** sodium–proton exchange, sperm, potassium channel, adenylate cyclase, cyclic AMP, fertilization, membrane hyperpolarization

## Abstract

Sperm capacitation is a complex process that takes place in the female reproductive tract and empowers mammalian sperm with the competence to fertilize an egg. It consists of an intricate cascade of events that can be mimicked *in vitro* through incubation in a medium containing essential components, such as bicarbonate, albumin, Ca^2+^, and energy substrates, among others. Genetic and pharmacological studies have underscored the unique significance of the K^+^ channel SLO3 in membrane potential hyperpolarization, as evidenced by the infertility of mice lacking its expression. Notably, two key molecular events, sperm hyperpolarization and intracellular alkalinization, are central to the capacitation process. SLO3 is activated by alkalinization. However, the molecular mechanisms responsible for intracellular alkalization and activation of SLO3 are not completely understood. In this study, we examined the impact of Na^+^/H^+^ exchangers (NHEs) on mouse sperm membrane hyperpolarization during capacitation. Pharmacological inhibition of the NHE1 blocked membrane hyperpolarization. A similar effect was observed in sperm deficient of the Ca^2+^ channel CatSper because of NHE1 not being activated by Ca^2+^. In addition, the sperm-specific NHE (sNHE) KO did not show membrane hyperpolarization upon capacitation or induction with cAMP analogs. Our results show that sNHE is dually modulated by cAMP and membrane hyperpolarization probably through its cyclic nucleotide–binding domain and the voltage-sensor motif, respectively. Together, sNHE and NHE1 provide the alkalinization need for SLO3 activation during capacitation.

The sperm capacitation process in mammalian sperm involves a complex cascade of events that unfolds within the female reproductive tract upon ejaculation (for a review, see Ref. (1)). These events are vital for enabling sperm to successfully fertilize the egg and are replicated *in vitro* using a specialized capacitating media. These media contain various components, including energy sources, bicarbonate, Ca^2+^, and albumin. At the molecular level, capacitation is hallmarked by critical processes, such as sperm membrane potential (*E*m) hyperpolarization and intracellular alkalinization (2, 3, 4). Genetic and pharmacological investigations have unveiled specific transporters that underlie the ionic fluxes involved in such changes. Among them, the atypical sperm-specific Na^+^/H^+^ exchanger (NHE; SLC9C1 aka sNHE) and the SLO3 K^+^ channel have emerged as key players. Their indispensability for capacitation is underscored by the fact that mice deficient in these genes exhibit infertility, albeit without discernible abnormalities in other physiological aspects, as these proteins are uniquely expressed in sperm (2, 5, 6). Given the importance of sperm *E*m hyperpolarization associated to SLO3 currents, it is intriguing that the molecular events leading to SLO3 opening have not been completely revealed yet, besides knowing that SLO3 responds to alkalinization.

The principal membrane H^+^ transporters responsible for mediating H^+^ efflux in response to the rising extracellular pH within the female reproductive tract are NHEs (for a review, see Ref. (7)). Three groups identify NHE isoforms in mammalian sperm: NHE1 and NHE5 (SLC9A subgroup), NHA1 and NHA2 (SLC9B subgroup), and sNHE as a member of the SLC9C subgroup, with the recent identification of SLC9C2 in at least rat and human sperm (8, 9). Their roles and significance in sperm physiology remain an active area of research.

The localization of NHE1 to the sperm midpiece is established (10), but its precise contribution to sperm fertility has been somewhat enigmatic. Notably, the breeding outcomes of NHE1-deficient mice have provided intriguing insights. While mating between *Nhe1* KO males and females yielded no successful breeding, a litter could be carried to term when *Nhe1*^+/−^ (HET) males mated with *Nhe1* KO females (11). As for NHE5, its functional importance in sperm physiology is yet to be elucidated, though it has been also localized to the midpiece of mouse sperm (10). Disruptions in the NHA exchangers provoked subfertility in single *Nha1* or *Nha2* KO males, whereas double KO males were rendered completely infertile, marked by severely compromised sperm motility. These phenotypes were associated with attenuated cAMP synthesis by soluble adenylyl cyclase (sAC) and reduced expression of the full-length sAC isoform (12). Addition of cell-permeable cAMP analogs rescued sperm motility defects, whereas fertility defects seemed to arise from deficient acrosome reaction (13). Regarding sNHE, it is localized to the principal piece of the sperm flagellum. It was found to affect sperm motility, a phenotype amenable to rescue through the addition of cell-permeable cAMP analogs (6). The presence of a cyclic nucleotide–binding domain (CNBD) and a putative voltage-sensor motif on sNHE suggests its potential regulation by cyclic nucleotides and changes in *E*m. It has been suggested that cAMP modulates intracellular pH (pHi) by regulating sNHE activity (14, 15). While these advances have broadened our understanding of key cellular processes that orchestrate capacitation, many questions remain unanswered regarding the precise physiological roles of these transporters (and even their presence) in sperm function.

In this article, we investigate the role of cAMP and its downstream targets in sperm alkalinization that drives hyperpolarization of *E*m during capacitation. Unexpectedly, inhibition of PKA catalytic activity did not impair *E*m hyperpolarization despite sAC activity being necessary for this change in *E*m. Noncapacitated sperm exposed to cAMP permeable analogs underwent *E*m hyperpolarization. Our results using a battery of genetic mouse models and pharmacology demonstrate the critical role of NHE1 and sNHE in the pathway leading to *E*m hyperpolarization, through pH modulation by Ca^2+^ and cAMP, respectively.

## Results

### PKA catalytic activity is not required for *E*m hyperpolarization

**Figure 1:**
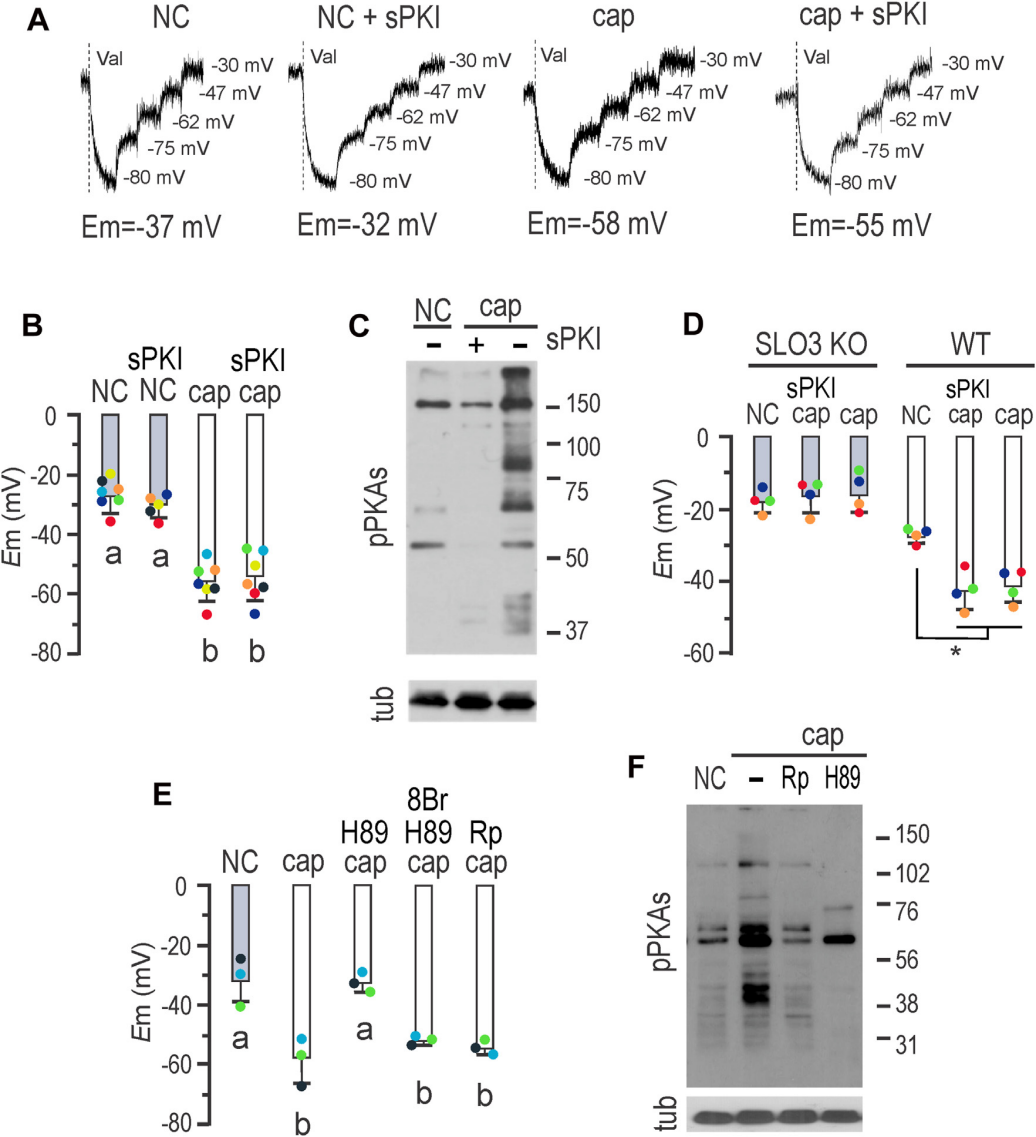
**Mouse sperm *E*m hyperpolarization can dispense PKA catalytic activity.***A*, fluorescence traces showing the values of the sperm *E*_m_ obtained after sperm incubation in either noncapacitating (NC) or capacitating (cap) conditions containing or not 15 μM sPKI for 60 min. Each experiment displays its calibration curve and the estimated *E*m value. *B*, summary of *E*_m_ measurements of sperm incubated in conditions depicted in *A* (mean ± SD; *n* ≥ 5). One-way ANOVA with Tukey’s multiple comparisons test was performed; *different letters* indicate statistically significant differences (*p* < 0.001). *C*, sperms were incubated for 60 min in NC or cap medium containing or not 15 μM sPKI. Each condition was processed for Western blot analysis with a monoclonal anti-pPKAs antibody. Membrane was stripped and analyzed for the presence of tubulin using anti-β-tub (clone E7). *D*, sperm *E*_m_ measurements obtained after 60 min incubation of either *Slo3* KO (*gray boxes*) or WT sperm (*white boxes*) in either NC or cap medium containing or not 15 μM sPKI. One-way ANOVA with Tukey’s multiple comparisons test was performed (mean ± SD; *n* = 4; ∗*p* < 0.001). Each *colored dot* represents the value for each independent sample. *E*, sperm *E*_m_ measurements obtained after 60 min incubation in NC or cap medium; as indicated, capacitating media were supplemented with either 30 μM H-89, 500 μM 8Br-cAMP (8Br), or 500 μM Rp-cAMPS (Rp). One-way ANOVA with Tukey’s multiple comparisons test was performed (mean ± SD; *n* = 3); *different letters* indicate statistically significant differences (*p* < 0.001). Each *colored dot* represents the value for each independent sample. *F*, sperms were incubated for 60 min in NC or cap medium; as indicated, cap media were supplemented with either 30 μM H-89 or 500 μM Rp. Each condition was processed for Western blot analysis with a monoclonal anti-pPKAs antibody. Membrane was stripped and analyzed for the presence of tubulin using anti-β-tub (clone E7).

**Supplemental Fig. S1:**
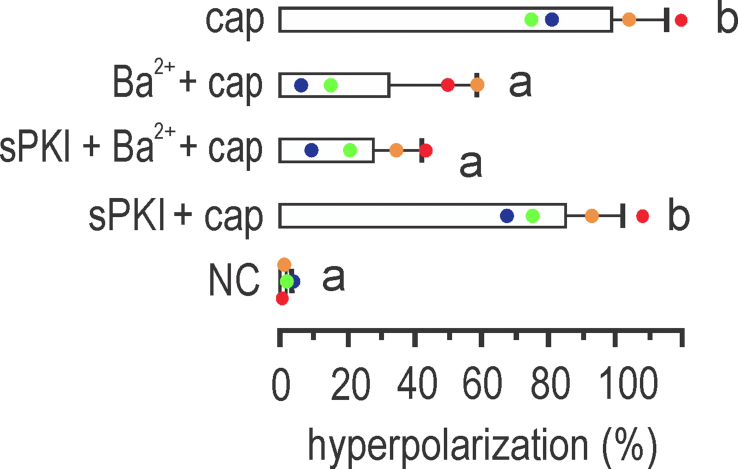
**Ba^2+^ blocks *E*m hyperpolarization even in the presence of sPKI.** Sperm *E*_m_ measurements obtained after incubation for 60 min in capacitating conditions containing either 1 mM BaCl_2_ or 15 μM sPKI or a combination of both. Results are expressed as a normalization of percentage of hyperpolarization considering mean NC and cap values as 0% and 100%, respectively (mean ± SD; n = 4); one-way ANOVA with Turkey’s multiple comparisons test was performed; different letters indicate statistically significant differences (*p* < 0.05).

One of the initial events in the capacitation-signaling pathway involves the bicarbonate-induced stimulation of sAC, leading to increased cAMP synthesis, which activates PKA (16). Two pieces of evidence support PKA's role in mouse sperm *E*m hyperpolarization: (1) the PKA inhibitor H-89 prevented *E*m hyperpolarization, when present during capacitation, and (2) *E*m hyperpolarization increased with bicarbonate in a concentration-dependent manner (17). To gain insights into this pathway, we attempted to inhibit *E*m hyperpolarization using the synthetic and permeable PKA inhibitor peptide sPKI, known to effectively and specifically inhibit its catalytic activity (18). Surprisingly, sperm *E*m hyperpolarization associated to capacitation was not inhibited by sPKI (Fig. 1, *A* and *B*). As a control, we show that sPKI did not affect *E*m in NC sperm (Fig. 1, *A* and *B*). We verified the effective blockade of PKA activity by sPKI through immunoblotting analysis using antibodies against phosphorylated PKA substrates (pPKAs) (19) (Fig. 1*C*). Furthermore, *E*m in capacitating medium containing sPKI depended on SLO3 activity, as cells did not hyperpolarize in the presence of BaCl_2_ (Fig. S1), an effective blocker of SLO3 currents (20). As an additional control, sperm from *Slo3* null mice did not hyperpolarize in the presence of sPKI, further disregarding the possibility of nonphysiological hyperpolarization caused by sPKI (Fig. 1*D*). To resolve this controversy regarding the role of PKA, we assayed the PKA inhibitors H-89 and Rp-cAMPS. Rp-cAMPS is a nonhydrolyzable and cell-permeable analog of cAMP that functions as a selective inhibitor of PKA (21). Figure 1*E* shows that while 30 μM H-89 inhibited *E*m hyperpolarization, it was restored by 500 μM 8Br-cAMP. Thus, this indicates that although H-89 impairs the onset of Em hyperpolarization, its mechanism of action is not through PKA inhibition, as a permeable analog of cAMP bypasses this inhibition. In addition, Rp-cAMPS did not inhibit *E*m hyperpolarization, whereas it inhibited PKA, as shown in the Western blot of Figure 1*F*.

### cAMP drives *E*m hyperpolarization

**Figure 2:**
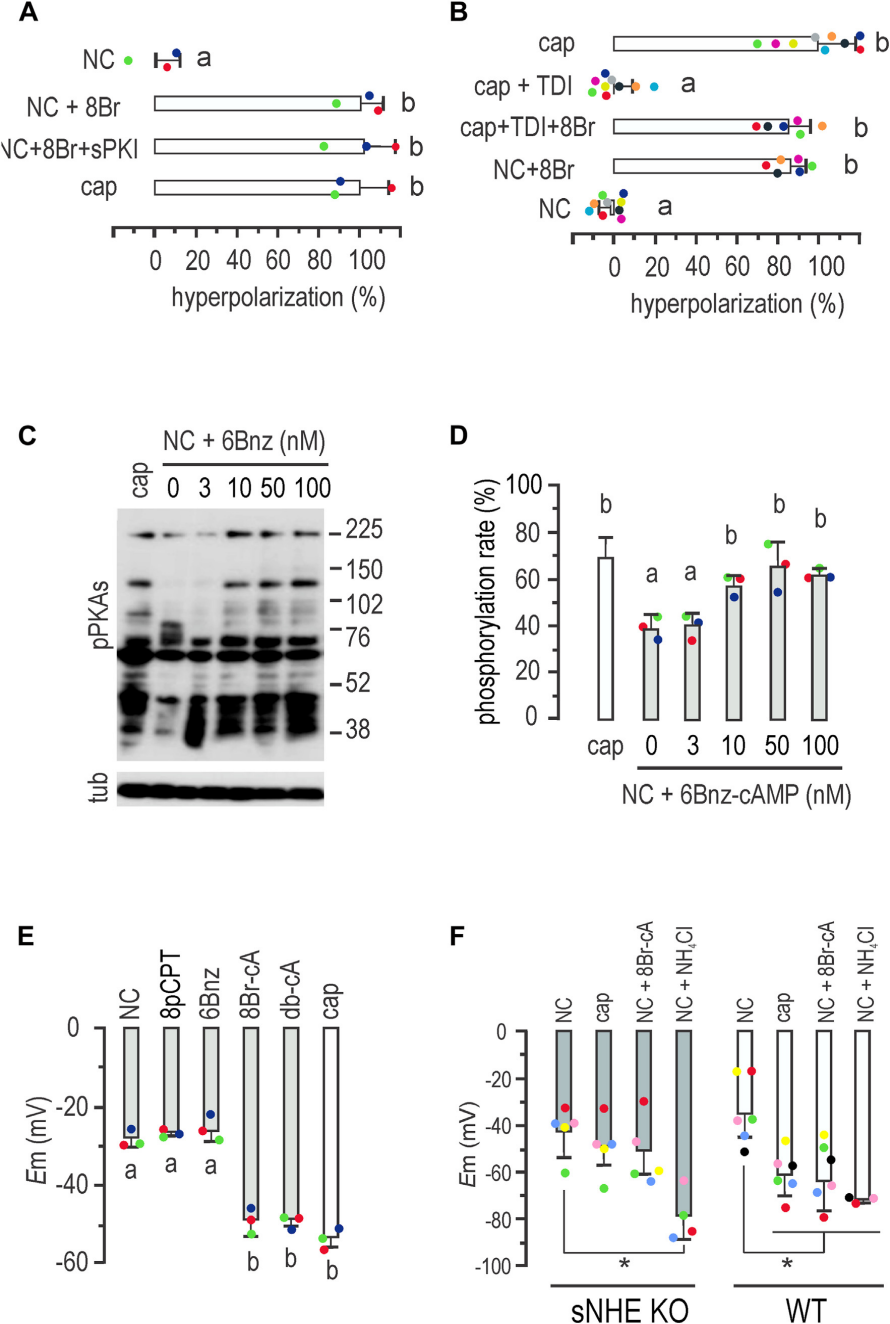
**Mouse sperm *E*m hyperpolarization is cAMP regulated and soluble Na**^**+**^**/H**^**+**^**exchanger (sNHE) dependent.***A*, sperm *E*_m_ obtained after incubation in either noncapacitating (NC) or capacitating (cap) conditions containing 500 μM 8Br-cAMP alone or in addition to15 μM sPKI for 60 min. Results are expressed as a normalization of percentage of hyperpolarization considering mean NC and cap values as 0% and 100%, respectively (mean ± SD; *n* = 4). One-way ANOVA with Tukey’s multiple comparisons test was performed; *different letters* indicate statistically significant differences (*p* < 0.001). *B*, sperm *E*_m_ obtained after incubation in either cap (with either 10 μM TDI10229 alone or in combination with 500 μM 8Br-cAMP) or NC conditions containing or not 500 μM 8Br-cAMP for 60 min. Results are expressed as a normalized percentage of hyperpolarization considering NC mean and cap mean values as 0% and 100%, respectively (mean ± SD; *n* = 9). One-way ANOVA with Tukey’s multiple comparisons test was performed. *Different letters* indicate statistically significant differences (*p* < 0.001). Each *colored dot* represents the value for each independent sample. *C*, sperms were incubated for 60 min in NC media containing increasing concentrations of 6Bnz-cAMP, as indicated. Each condition was processed for Western blot analysis with a monoclonal anti-pPKAs antibody. The membrane was stripped and analyzed for the presence of tubulin using anti-β-tub (clone E7). *D*, summary of densitometry analysis of sperm cells incubated as in *A*. One-way ANOVA with Tukey’s multiple comparisons test was performed (mean ± SD; *n* = 3). *Different letters* indicate statistically significant differences (*p* < 0.05). *E*, *E*_m_ measurements of sperm incubated in NC conditions containing either 30 μM 8-pCPT-2′-O-Me-cAMP (8pCPT), 50 nM 6Bnz-cAMP, 500 μM 8Br-cAMP, or 500 μM dibutyryl-cAMP (db-cAMP) for 60 min (mean ± SD; *n* = 3). One-way ANOVA with Tukey’s multiple comparisons test was performed; different letters indicate statistically significant differences (*p* < 0.001). *F*, summary of *E*_m_ sperm measurements from *sNhe* KO (*gray boxes*) or WT mice (*black boxes*) obtained after incubation in either cap or NC conditions containing 500 μM 8Br-cAMP or 10 mM NH_4_Cl for 60 min. One-way ANOVA with Tukey’s multiple comparisons test was performed (mean ± SD; *n* = 5; ∗*p* < 0.05).

Figure 1 showed that while H-89 impaired the onset of *E*m hyperpolarization, this blockade was bypassed by the permeable cAMP analog 8Br-cAMP, known to stimulate phosphorylation of PKAs (22). Thus, we aimed at analyzing the effect of cAMP on *E*m hyperpolarization. In these experiments, it should be noted that each subset of data has its own controls, for ensuring that all reagents are working as expected. When NC media were supplemented with 8Br-cAMP, *E*m hyperpolarization was observed, while this effect was not inhibited by sPKI (Fig. 2*A*). These results, together with the effect of H-89, indicate that while sPKI, Rp-cAMPS, and H-89 inhibit PKA, H-89 exerts an additional nonspecific effect, which is overridden by addition of a permeable cAMP analog. We further studied the effect of cAMP for the onset of *E*m hyperpolarization, through inhibition of cAMP production during capacitation using the potent and selective sAC inhibitor TDI-10229 (23). As shown in Figure 2*B*, TDI-10229 prevented *E*m hyperpolarization when added to capacitating media. The addition of 8Br-cAMP overcame the inhibitory effect of TDI-10229, further supporting the role of cAMP in *E*m hyperpolarization, independently of PKA catalytic activity.

### cAMP targets during promotion of *E*m hyperpolarization

Results presented in Figures 1 and 2 indicated that cAMP promotes *E*m hyperpolarization, independently of PKA catalytic activity. The second messenger cAMP has, besides PKA, two other known effectors: the exchange protein activated by cAMP (EPAC, a guanine-nucleotide-exchange factor) and the CNBD found in cyclic nucleotide–gated ion channels (for a comprehensive review, see Ref. (1)). To shed light on the signaling pathway through which cAMP promotes sperm *E*m hyperpolarization, we selected specific agonists of cAMP targets, including the widely used 8-pCPT-2′-O-Me-cAMP to activate EPAC and the membrane-permeant PKA selective agonist N6-benzyladenosine-cAMP (6Bnz-cAMP). To select an appropriate concentration of 6Bnz-cAMP, we initially exposed sperm to increasing concentrations of the PKA agonist for 60 min in noncapacitating media to assess PKA activity, as indicated by the *in vivo* phosphorylation of PKAs. Western blots using anti-pPKAs antibodies revealed that a concentration of 50 nM 6Bnz-cAMP induced a saturating phosphorylation of PKAs (Fig. 2, *C* and *D*) and used hereafter to stimulate PKA. Then, the roles of EPAC and PKA in *E*m were then addressed by direct stimulation. As previously shown, 50 μM 8-pCPT-2′-O-Me-cAMP was used for direct stimulation of EPAC (24). While both general cAMP analogs db-cAMP and 8Br-cAMP promoted *E*m hyperpolarization, specific activation neither of EPAC with 8-pCPT-2′-O-Me-cAMP nor of PKA with 6Bnz-cAMP induced this *E*m shift (Fig. 2*E*), ruling out the direct involvement of EPAC and PKA in this process.

**Figure 3:**
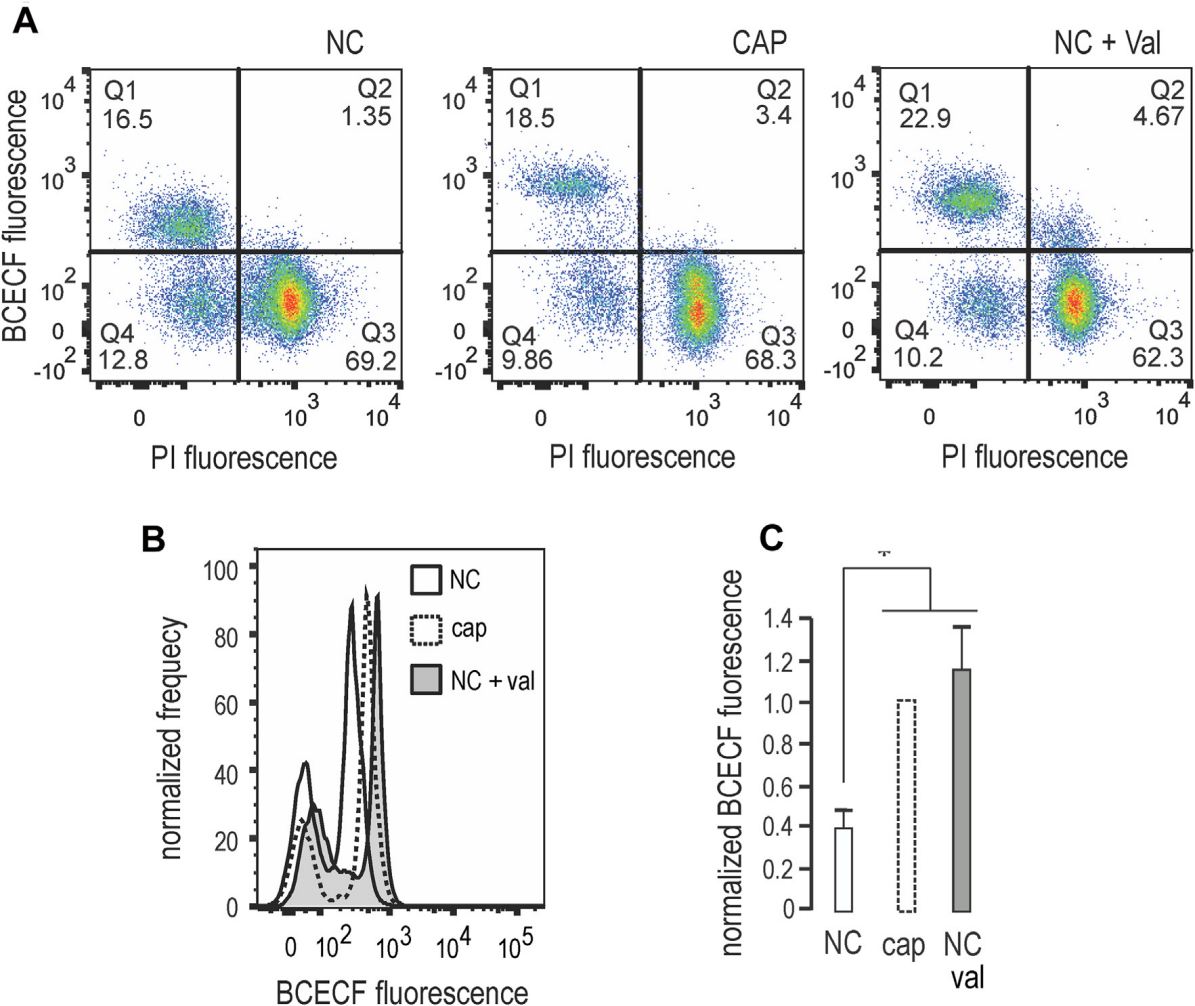
***E*m hyperpolarization induces intracellular alkalinization.***A*, sperms were incubated in capacitating (cap) or noncapacitating (NC) media either in the absence (NC) or the presence of 1 μM valinomycin (NC + Val). Representative BCECF (pH-sensitive fluorophore) *versus* propidium iodide (PI) two-dimensional fluorescence dot plot analysis. *Squares* marked as Q1 (*upper left squares*) define sperm with high intracellular pH displaying membrane integrity (low PI staining). *Numbers* in each *square* indicate percentage of total population detected. *B*, sperms were incubated in cap or NC media either in the absence (NC) or the presence of 1 μM valinomycin (NC + Val). The two-dimensional dot plots shown in *A* were used to select sperm with low (live) PI staining. The histogram analysis depicts normalized frequency of sperm and BCECF fluorescence of live sperm populations. *C*, normalized median fluorescence intensity of BCECF compared to the control capacitating condition (mean ± SD, n = 4, ∗*p* < 0.05).

Over 20 years ago, the sNHE/NHE10 (SLC9C1) has been shown to be essential for mice’s male fertility (25), expressed at the principal piece. A unique feature of sNHE/NHE10 (SLC9C1) is the possession of a voltage-sensor domain (found in voltage-gated channels), and a CNBD at cytosolic region. Exchangers of the NHE family regulate pHi, while SLO3 channels are activated by alkalinization. Thus, the presence of a CNBD in Snhe, added to the role of cAMP driving hyperpolarization, led us to hypothesize that sNHE might be the target of cAMP. As pharmacological inhibitors of sNHE are not yet available, we analyzed *E*m hyperpolarization in sperm from sNHE-null mice. Figure 2*F* demonstrates that sperm from *sNhe* KO mice did not undergo hyperpolarization when incubated in capacitating conditions for 60 min. Gain of function was not observed in the presence of the agonist 8Br-cAMP, which could be probably attributed to the absence of the CNBD harbored by sNHE. Alkalinization induced by NH_4_Cl addition promoted *E*m hyperpolarization in both WT and sNHE KO sperm, confirming the functional response of SLO3 channels in this KO model. Interestingly, previous research showed that the sAC inhibitor TDI-10229 blocked pHi increase associated with capacitation (23). As mentioned, sNHE is proposed to be also regulated by *E*m through a voltage-sensor domain, as generally found in voltage-gated channels (26). Thus, *E*m hyperpolarization could be part of a positive feedback loop, increasing the activity of sNHE. To address this hypothesis, we analyzed the effect of *E*m hyperpolarization on pHi, through stimulation with the K^+^ ionophore valinomycin, promoting a pharmacological hyperpolarization (27). Noncapacitated sperm incubated with valinomycin showed an increase of pHi similar to that observed in capacitated cells, as evidenced by fluorescence increase of BCECF-loaded sperm (Fig. 3, *A*–*C*), in agreement with previous results (28). Of note, this effect on pHi was absent in sNHE-deficient mice (28).

### Role of NHEs in sperm *E*m hyperpolarization

**Figure 4:**
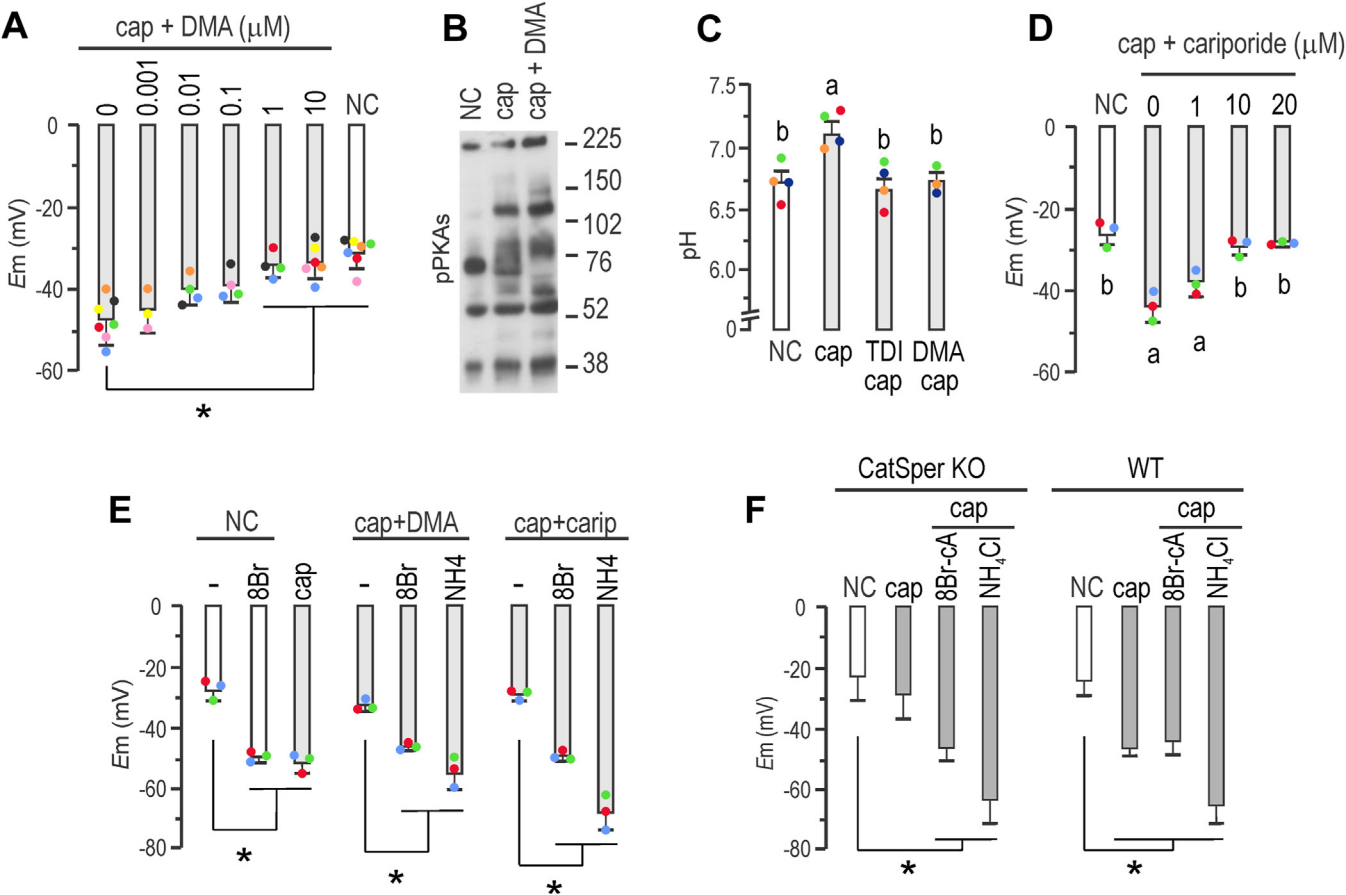
**NHE1 activity through Ca**^**2+**^**stimulation is conducive to *E*m hyperpolarization.***A*, *E*_m_ obtained after sperm incubation in either noncapacitating (NC) or capacitating (cap) conditions containing different concentrations of 5-(*N*,*N*-dimethyl)-amiloride (DMA) as indicated, for 60 min. One-way ANOVA with Tukey’s multiple comparisons test was performed (mean ± SD; *n* = 4; ∗*p* < 0.001). *B*, sperms were incubated for 60 min in NC or in cap medium in the presence or the absence of 10 μM DMA. Each condition was processed for Western blot analysis with a monoclonal anti-pPKAs antibody. *C*, sperms were incubated for 60 min in NC or cap medium containing or not 10 μM TDI-10229 (TDI) or 1 μM DMA. One-way ANOVA with Dunnett’s multiple comparisons test was performed; *different letters* indicate statistically significant differences (*p* < 0.001). *D*, *E*_m_ obtained after sperm incubation in either NC or cap conditions containing different concentrations of cariporide, as indicated, for 60 min. One-way ANOVA with Tukey’s multiple comparisons test was performed; *different letters* indicate statistically significant differences (mean ± SD; *n* = 4; ∗*p* < 0.001). *E*, *E*_m_ obtained after sperm incubation in NC conditions in the presence or not of 500 μM 8Br-cAMP (8Br) or in cap conditions. As specified, cap conditions were supplemented with either 1 μM DMA or 10 μM cariporide for 60 min. As indicated, these conditions were also supplemented with 500 μM 8Br-cAMP or 10 mM NH4Cl. One-way ANOVA with Tukey’s multiple comparisons test was performed (mean ± SD; *n* = 4; ∗*p* < 0.01). *F*, sperm *E*_m_ from either *CatSper1* KO (*left panel*) or WT mice (*right panel*) were obtained after sperm incubation in either NC (with *boxes*) or cap conditions (*gray boxes*) containing or not either 500 μM 8Br-cAMP or 10 mM NH_4_Cl for 60 min. One-way ANOVA with Tukey’s multiple comparisons test was performed (mean ± SD; *n* = 5; ∗*p* < 0.005).

sNHE is not the sole exchanger present in mouse sperm. To explore the role of other NHEs in *E*m hyperpolarization, we employed the potent inhibitor 5-(*N*,*N*-dimethyl)-amiloride (DMA), which targets members of the SLC9A subfamily, including NHE1, NHE2, and NHE3, with K_i_ values of 0.02, 0.25, and 14 μM, respectively, and negligible effects on NHE4, NHE5, and NHE7 (29). DMA, which does not directly affect SLO3 or CatSper channels as recently shown (30), was added at different concentrations to capacitating media to address its effect on *E*m hyperpolarization. Figure 4*A* shows a concentration-dependent inhibition of *E*m hyperpolarization. A concentration of 1 μM DMA significantly inhibited hyperpolarization. Of note, 10 μM DMA did not affect the phosphorylation of PKAs (Fig. 4*B*), thus excluding an effect on cAMP synthesis. Considering that NHE1, NHE5, NHA1, NHA2, and sNHE are known to be present in mouse sperm plasma membrane, and that DMA inhibits NHE1, NHE2, and NHE3, then NHE1 might be the primary target of DMA in these cells (29). In line with these findings, recent research demonstrated that DMA reduced K^+^ currents in mouse sperm by impairing the regulation of pHi (30). Along this line, Figure 4*C* shows that DMA inhibited alkalinization associated to capacitation in mouse sperm.

A second inhibitor, cariporide, which selectively targets NHE1 (31), was also tested. Figure 4*D* shows that it effectively inhibited *E*m hyperpolarization when added to capacitating media at 10 μM. The effects of both cariporide and DMA could be bypassed by the addition of either 8Br-cAMP that directly stimulates sNHE (not inhibited by any NHE inhibitor) or NH_4_Cl that directly stimulates SLO3 (Fig. 4*E*).

**Figure 5:**
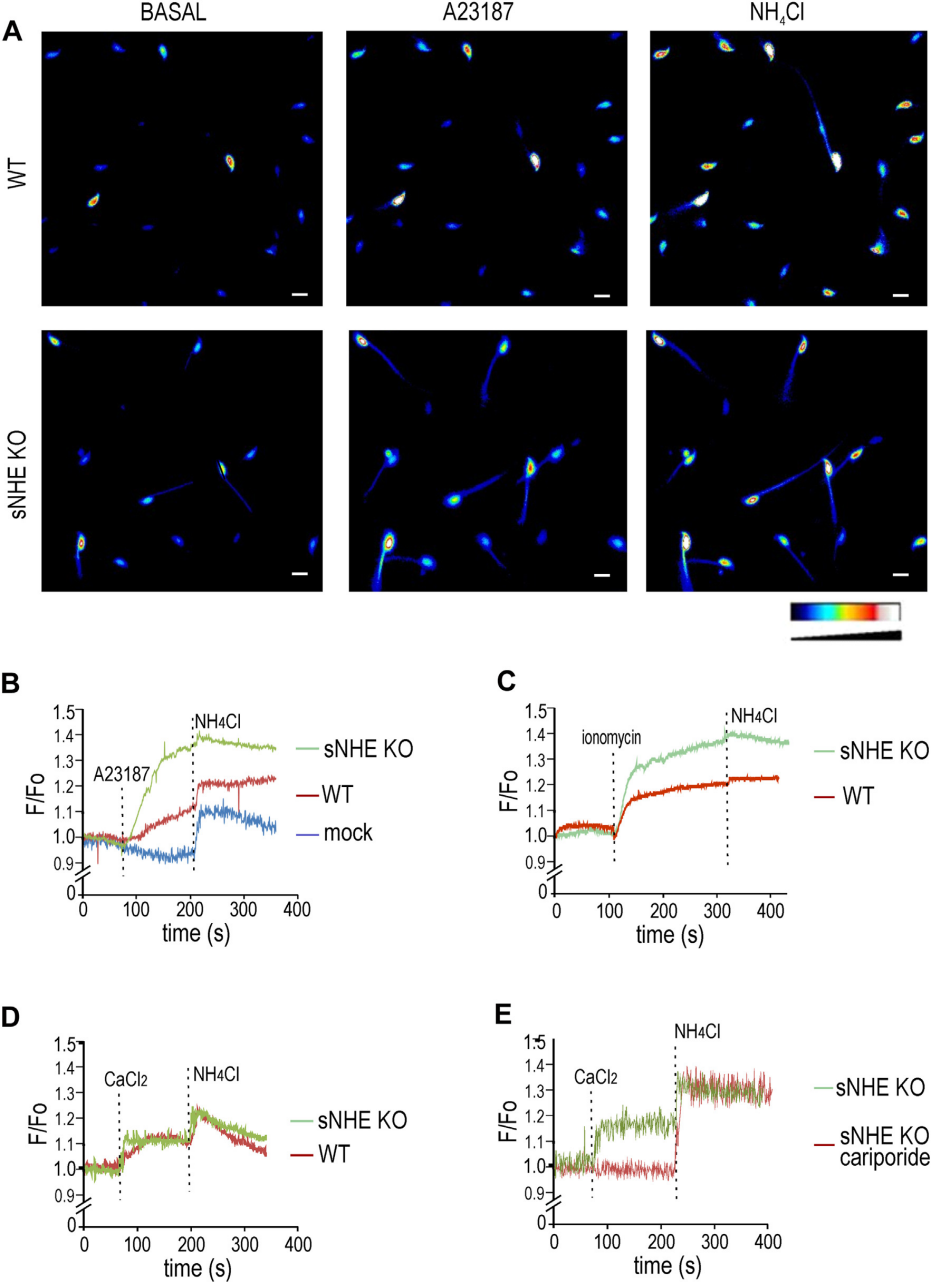
**Intracellular Ca**^**2+**^**increase promotes cytoplasmic alkalinization in sperm cells.** Noncapacitated (NC) sperm cells were loaded with 0.5 μM BCECF-AM for 30 min before smearing onto laminin-precoated coverslips to record fluorescence. *A*, representative fluorescence images of WT (*upper panels*) and *sNhe* KO (*lower panels*) sperm exposed to 10 μM of the ionophore A23187, followed by 10 mM NH_4_Cl. Reference bar for fluorescence intensity is depicted. Scale bar represents 10 μm. *B*, summary average traces of experiments performed in *A*, including a mock treatment on WT sperm performed with dimethyl sulfoxide instead of A23187. About 122 of 140 cells and 77 of 99 cells analyzed responded in WT and sNHE KO, respectively; n = 5. *C*, summary average traces of either WT or *sNhe* KO sperm were exposed to 10 μM ionomycin followed by 20 mM NH_4_Cl. About 117 of 147 cells and 92 of 110 cells analyzed responded in WT and sNHE KO, respectively; n = 4. *D*, summary average traces of either WT or *sNhe* KO sperm incubated in nominal zero Ca^2+^ (no added Ca^2+^ salts) challenged with 1.7 mM CaCl_2_ and followed by 20 mM NH_4_Cl. About 106 of 139 cells (n = 4) and 69 of 94 cells (n = 3) analyzed responded in WT and sNHE KO, respectively. *E*, summary average traces of *sNhe* KO sperm incubated in nominal zero Ca^2+^ (no added Ca^2+^ salts) in the presence or not of 10 μM cariporide, and challenged with 1.7 mM CaCl_2,_ followed by 20 mM NH_4_Cl. About 78 of 101 cells (n = 3) and 20 of 83 cells (n = 3) analyzed responded in the absence or the presence of cariporide, respectively. sNHE, soluble Na^+^/H^+^ exchanger.

NHE1 possesses a C-terminal domain with extensive disordered regions (32), which binds calmodulin in the presence of Ca^2+^ (33). In other cell types, this binding induces an alkaline shift in the pHi sensitivity of NHE1, resulting in its activation at less acidic pHi (34). Therefore, the increase in intracellular Ca^2+^ associated with capacitation could activate NHE1, leading to an increase in pHi. To investigate the role of Ca^2+^ in *E*m hyperpolarization, we assessed *E*m in the CatSper1 KO model. Figure 4,*F* demonstrates that sperm from CatSper1 KO mice exhibited deficient *E*m hyperpolarization, which could be restored by the addition of either 8Br-cAMP or NH_4_Cl, similar to the effects observed when inhibiting NHE1 by either DMA or cariporide (Fig. 4*E*). Accordingly, Figure 5, *A* and *B* shows that the Ca^2+^ ionophore A23187 promoted pHi increase in sperm loaded with the pH-sensitive dye BCECF. Treatment with Ca^2+^ ionophore A23187 promoted a pH increase also in sNHE KO but to a greater extent in sNHE KO than in WT sperm. Although it is speculative, this could be attributed to overexpression of NHE1 as a compensatory effect in the sNHE KO model. As a control, NH_4_Cl was used to evoke pHi increase in both WT and sNHE KO sperm. Same effect was observed when sperms were challenged with ionomycin, as a second Ca^2+^ ionophore (Fig. 5*C*). Even though Ca^2+^ ionophores are used to increase intracellular Ca^2+^, it can be argued that they also promote pharmacological pHi increase because of H^+^ extrusion, as ionophores exchange Ca^2+^ for H^+^. Thus, as a second approach, cells were incubated in media without added Ca^2+^ salts (which still contain micromolar concentrations of Ca^2+^ (35)) and challenged with 1.7 mM Ca^2+^ (Fig. 5*D*). As before, pHi increase could be clearly evidenced, both in WT and sNHE KO sperm, further substantiating the role of Ca^2+^ on pHi. To further address the role of NHE1 as the target of intracellular Ca^2+^ increase, we incubated sperm from sNHE KO mice with the NHE1 inhibitor cariporide, showed to impair *E*m hyperpolarization (Fig. 4*E*). As shown in Figure 5*E*, cariporide impairs intracellular alkalinization promoted by addition of CaCl_2_ to a Ca^2+^ zero medium. Altogether, these results support the role of Ca^2+^ in the pathway to hyperpolarization and shed light onto the lack of hyperpolarization in CatSper KO sperm.

These findings indicate that cAMP is responsible for *E*m hyperpolarization, which includes an increase in pH_i_ mediated by NHEs, independent of PKA catalytic activity, and ultimately promoting SLO3 channel opening.

## Discussion

The mammalian sperm–specific K^+^ channel, SLO3, plays a pivotal role in processes leading to sperm capacitation. *Slo3* KO mice are unable to undergo a physiologically stimulated acrosome reaction and are consequently infertile (2, 36), underscoring the significance of investigating sperm *E*m hyperpolarization. However, the regulation of this channel during capacitation remains poorly understood.

Experiments conducted by Escoffier *et al.* (37) demonstrated that *E*m hyperpolarization associated to capacitation was inhibited by H-89. It is worth noting that H-89 is now recognized for its nonspecific effects (38). As shown herein, the inhibition exerted by H-89 was overridden by addition of 8Br-cAMP, excluding the role of PKA in this pathway. On the other hand, synthetic short peptides of PKI, such as PKI-(14–22)-amide (sPKI), have gained wide acceptance as pharmaceutical agents for selectively inhibiting PKA activity (39), demonstrating a high level of specificity. Our observations showed that sperm capacitated in the presence of either sPKI or the cell-permeable analog of cAMP Rp-cAMPS that functions as a selective PKA inhibitor, while displaying inhibition of PKAs phosphorylation, exhibited hyperpolarized *E*m. Furthermore, inhibition of sAC by TDI-10229 (23) blocked *E*m hyperpolarization, consistent with its impact on impairing pHi alkalinization (23). When the permeable analog 8Br-cAMP was introduced to a noncapacitated sperm, it induced *E*m hyperpolarization, a finding that aligns with previous reports involving a different cAMP analog (37). This supports the role of cAMP in the road of activating SLO3 channels and excludes PKA as indispensable.

The mouse SLO3 channel was found to be activated by intracellular alkalinization (40), though the precise mechanism of its modulation by pHi remains unresolved (for an in-depth review, see Ref. (41)). An important regulator of SLO3 is the leucine-rich-repeat–containing protein 52 (LRRC52) (42). The significance of LRRC52 for SLO3 activity was demonstrated in *Lrrc52* KO mice, where alkalinization failed to hyperpolarize sperm *E*m to the same extent as in WT sperm, suggesting a crucial role for LRCC52 in SLO3 response to alkalinization (41). In this regard, NHEs have emerged as potential contributors to sperm alkalinization during capacitation. NHEs are responsible for regulating the pH of different cell compartments in a variety of cell types. Of particular importance in sperm physiology, sNHE is located in the principal piece of the sperm flagellum and possesses a CNBD (25). Sperm from sNHE-null mice did not undergo capacitation-associated hyperactivation (6), although this phenotype was restored by the addition of permeable cAMP analogs (43). Similar to slc9c1 KO, disruption of either NHA1 or NHA2 resulted in a reduced sperm motility phenotype, which was also rescued by incubating sperm with cAMP analogs, pointing toward the role of NHEs in proper regulation of sAC activity and/or expression (12). However, our findings herein demonstrate that cAMP analogs did not restore *E*m hyperpolarization in sNHE-null sperm. Despite the ability of cAMP addition to restore motility in sNHE-null sperm (6, 25), it did not reinstate *E*m hyperpolarization, indicating the involvement of sNHE in this pathway. Although it awaits further demonstration, the CNBD present in sNHE is probably the target of cAMP, driving sNHE activity (25). Recently, a human case was reported in which a mutation in sNHE resulted in a deletion in the CNBD. These sperm, lacking a functional CNBD, exhibited asthenozoospermia and infertility, indicating its importance in human sperm (44).

Physiological modulation of the NHE family has been a subject of study for many years, mainly through pharmacology. Modified analogs of amiloride were designed to enhance specificity toward NHEs. DMA bears a double substitution of the 5-amino group nitrogen, which increases its potency and selectivity toward NHE1. Our results demonstrated that DMA produced a robust inhibition of *E*m hyperpolarization when present in capacitating media. Cariporide, a nonrelated amiloride inhibitor of NHE1, with negligible effects on either Na^+^/Ca^2+^ exchangers or ENaCs (45), also inhibited *E*m hyperpolarization. In all instances, *E*m hyperpolarization could be reinstated by the addition of permeable cAMP analogs. Therefore, these results indicate the participation of both sNHE and NHE1 in triggering *E*m hyperpolarization of mouse sperm. cAMP could possibly act through the CNBD present in sNHE to increase pHi, triggering the opening of SLO3. This *E*m hyperpolarization, in turn, could participate in a positive feedback loop onto sNHE, as previously suggested (26, 28). On the other hand, intracellular Ca^2+^ might activate NHE1 through its extensive disordered regions in the long cytoplasmic C-terminal domain (32, 33). It has been proposed that this binding allows activation of NHE1 at a less acidic pHi (34). Therefore, upon the increase in intracellular Ca^2+^ associated with capacitation, NHE1 could drive pHi to a more alkaline state. In human sperm, NHE1 has been recently shown to be expressed at low amounts (46). Thus, possible differences in the control of pHi between mouse and human can be expected.

**Figure 6:**
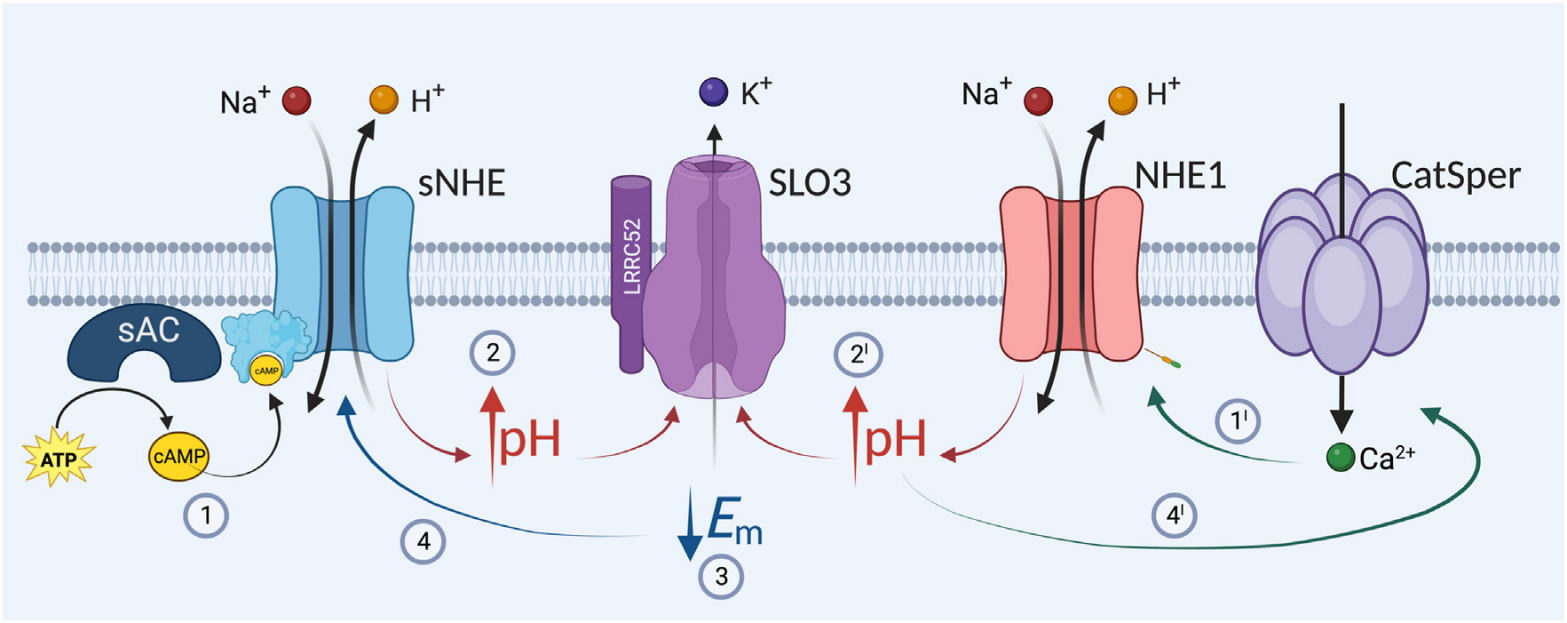
**Working model proposing a dual action of sNHE and NHE1 on sperm *E*m hyperpolarization.** (1) Synthesis of cAMP induces alkalinization *via* sNHE, which, together with the action of NHE1 driven by intracellular Ca^2+^ increase (1′), promote the necessary alkalinization (2 and 2′) to increase the conductance of SLO3 (3). In turn, *E*m hyperpolarization stimulates a positive feedback loop that further activates sNHE (4). It is worth noting that the sole action of sNHE (inhibition of NHE1) or of NHE1 (in the case of *sNhe* KO) is not sufficient, under physiological conditions, to promote SLO3 opening. sNHE, soluble Na^+^/H^+^ exchanger.

Considering these findings, we propose a mechanism that regulates pHi and *E*m hyperpolarization in mouse sperm, in which NHE1 and sNHE act synergistically (Fig. 6). Synthesis of cAMP induces alkalinization *via* sNHE, which, together with the action of NHE1 driven by intracellular Ca^2+^ increase, promotes the necessary alkalinization to increase the conductance of SLO3. In turn, the positive feedback loop where *E*m hyperpolarization further activates sNHE is also proposed and awaits further insight.

This work paves the way for the study of the role of NHEs in mammalian sperm, considering their pivotal role in capacitation. These insights into the regulatory network of sperm capacitation contribute to our understanding of the fundamental processes that underlie fertilization competence, offering new perspectives for future research in this field.

## Experimental procedures

### Experimental design

C57BL/6 male mature (10–13 weeks old) male mice (WT, *Catsper1* KO (5), *Slo3* KO (2) and *sNhe* KO (25)) were used. In all cases, mice housing and all experimental procedures were conducted in accordance with the corresponding Institutional Animal Care guidelines, reviewed and approved by the Ethical Committees of the Instituto de Biología y Medicina Experimental, Buenos Aires, Argentina #32/2021, Animal Care and Use Committee of the Facultad de Ciencias Bioquímicas y Farmacéuticas de Rosario (UNR), Argentina (#380/2023), and of the Instituto de Biotecnología, UNAM, Mexico. The Guide for Care and Use of Laboratory Animals approved by the National Institutes of Health was strictly met. In all cases, sperms were prepared as detailed later, using high-grade reagents, as follows: bovine serum albumin (BSA, fatty acid free), cariporide (HOE-642), isobutilmetilxantina, and 2′-O-dibutiril adenosín monofosfato-3′,5′ cíclico, carbonyl cyanide 3-chlorophenylhydrazone, dimethyl sulfoxide, Ca^2+^ ionophore A23187, and ionomycin were purchased from Sigma. PKI 14–22 amide myristoylated (sPKI) was obtained from Tocris. 3-Amino-*N*-(aminoiminomethyl)-6-chloro-5-(dimethylamino)-2-pyrazinecarboxamide monohydrochloride (DMA), 8-bromo-cyclic 3′,5′-(hydrogen phosphate)-adenosine monosodium salt (8Br-cAMP), *N*-benzoyl-adenosine cyclic 3′,5′-(hydrogen phosphate) (6Bnz-cAMP), monosodium salt, and 8-[(4-chlorophenyl)thio]-2′-O-methyl-adenosine cyclic 3′,5′-hydrogen phosphate (8pCPT-2-O′-methyl cAMP), monosodium salt, and valinomycin were purchased from Cayman Chemicals. Anti-pPKAs (clone 100G7E) antibodies and horseradish peroxidase–conjugated anti-mouse and anti-rabbit IgG were purchased from Cell Signaling Technology. β-tubulin (clone E7) was purchased by Developmental Studies Hybridoma Bank. The sAC inhibitor TDI-10229 was kindly provided by Drs Levin and Buck, Department of Pharmacology, Weill Cornell Medicine, New York City, USA. 3,3-Dipropylthiadicarbocyanine iodide (DiSC3(5)), BCECF-AM, and pluronic acid from Invitrogen, Thermo Fisher Scientific; while propidium iodide from Santa Cruz Biotechnology. DiSC3(5), BCECF-AM, and pluronic acid were dissolved in dimethyl sulfoxide; propidium iodide was dissolved in hexa-distilled water.

### Sperm preparation

Cauda epididymal mouse sperms were collected from adult male mice (10–13 weeks old). Each minced cauda epididymis was placed in 600 μl of Hepes-buffered TYH medium (H-TYH) containing 119.3 mM NaCl, 4.7 mM KCl, 1.2 mM KH_2_PO_4_, 1.2 mM MgSO_4_, 5.6 mM glucose, 0.5 mM sodium pyruvate, 1.7 mM Ca^2+^, and 20 mM Hepes (pH 7.3), accounting for H-TYH medium (“NC medium”). After 15 min of incubation at 37 °C (swim-out), epididymides were removed and the suspension was adjusted with NC medium to a final concentration of 1 to 2 × 10^7^ cells/ml. For capacitation, BSA and NaHCO_3_ were added to final concentrations of 5 mg/ml and 20 mM, respectively (“cap medium”) and incubated at 37 °C for at least 1 h, or the indicated period.

### SDS-PAGE and immunoblotting

After treatment, sperms were collected by centrifugation, washed in 1 ml of PBS, resuspended in Laemmli sample buffer without β-mercaptoethanol, and boiled for 5 min. After centrifugation, 5% β-mercaptoethanol was added to the supernatants and boiled again for 5 min. Protein extracts equivalent to 1 to 2 × 10^6^ sperm per lane were subjected to SDS-PAGE and electrotransferred to polyvinylidene fluoride membranes (Bio-Rad) at 250 mA for 60 min on ice. Membranes were blocked with 3% BSA in Tris-buffered saline (TBS) containing 0.1% Tween-20 (T-TBS). Antibodies were diluted in T-TBS containing 1% BSA as follows: 1/3,000 for anti-pPKAs and 1/10,000 for anti-β-tubulin. Secondary antibodies were diluted 1/10,000 in T-TBS and developed using an enhanced chemiluminescence detection kit (ECL Kallium Biolumina) according to the manufacturer’s instructions. When necessary, polyvinylidene fluoride membranes were stripped at 60 °C for 15 min in 2% SDS, 0.74% β-mercaptoethanol, and 62.5 mM Tris (pH 6.5), and washed six times, 5 min each time, in T-TBS. In all experiments, molecular masses were expressed in kilodaltons (kDas).

### Membrane potential assay in cell populations

Sperm *E*m changes were assessed using DiSC_3_(5), as previously described (47). After treatment, cells were loaded with 1 μM of the membrane-potential-sensitive dye DiSC_3_(5) (Molecular Probes) for 2 min. Sperms were transferred to a gently stirred cuvette at 37 °C, and the fluorescence was monitored with a Cary Eclipse fluorescence spectrophotometer at 620/670 nm excitation/emission wavelengths. Carbonyl cyanide 3-chlorophenylhydrazone (0.5 μM) was added as uncoupler of oxidative phosphorylation to avoid mitochondrial contribution to the recorded *E*m. Recordings were initiated when steady-state fluorescence was reached, and calibration was performed at the end of each measure by adding 1 μM valinomycin and sequential additions of KCl for internal calibration curves, as previously described (48). Sperm *E*m was obtained from the initial fluorescence (measured as Arbitrary Fluorescence Units) by linearly interpolating it in the theoretical *E*m values from the calibration curve against arbitrary fluorescence units of each trace. This internal calibration for each determination compensates for variables that influence the absolute fluorescence values.

### Determination of pHi by flow cytometry

Sperm pHi changes were assessed using BCECF-AM as previously described (49). After incubation in the appropriate medium, samples were centrifuged at 400*g* for 4 min at room temperature and resuspended in 200 μl of NC H-TYH medium containing 0.5 μM BCECF-AM for 20 min at 37 °C. Samples were washed again and resuspended in 50 μl of NC H-TYH medium. Before collecting data, 3 μM of propidium iodide was added to monitor viability. Data were recorded as individual cellular events using a MACSQuant Analyzer cytometer (Miltenyi Biotec). Side-scatter area and forward-scatter area (FSC-A) data were collected from 20,000 events per sample to define sperm population as previously described (17). In all cases, doublet exclusion was performed analyzing two-dimensional dot plot FSC-A *versus* FSC-H. Positive cells for BCECF were collected using fluorescein isothiocyanate filter (FITC; 530/30) together with peridinin chlorophyll protein complex (PerCP; 670LP) filter. Although the two indicators had minimal emission overlap, compensation was done. For calibration curves, samples were split and resuspended with high K^+^ buffered solutions at pH 6.3, 6.5, 7.0, 7.4, or 8.0 (1.2 mM MgSO_4_, 1.6 mM CaCl_2_, 23.8 mM Hepes, 2.78 mM glucose, 3.38 mM sodium pyruvate, and 120 mM KCl; pH previously adjusted with NaOH), and 5 μM nigericin was added to equilibrate intracellular and extracellular pH. Data were analyzed using FlowJo software (version 10.0.7; BD Biosciences).

### Analysis of pHi by single cell imaging

Sperms were loaded with the fluorescent pHi indicator as described for flow cytometry. Cells were later adhered to 1 mg/ml laminin-precoated coverslips, allowing their flagella to move continuously. The coverslip was mounted on a chamber (Harvard Apparatus) and placed on the stage of an inverted microscope (Eclipse TE 300; Nikon). Fluorescence illumination was supplied by a Luxeon V Star Lambertian Cyan LED (Lumileds Lighting LLC) attached to a custom-built stroboscopic control box. The LED was mounted into a FlashCube40 assembly with a dichroic mirror (M40-DC400; Rapp Opto Electronic; bandwidths: excitation, 450–490 nm; dichroic mirror, 505 nm; and emission, 520–560 nm). The LED output was synchronized to the Exposure Out signal of an iXon 888 CCD camera *via* the control box to produce a single flash of 2-ms duration per individual exposure. The camera exposure time was set equivalent to the flash duration (2 ms). Images were collected every 500 ms using iQ software (Andor Technology).

### Statistical analysis

Data are expressed as mean ± SD of at least three independent experiments for all determinations. Statistical analyses were performed using the GraphPad Prism 6 software (GraphPad Software, Inc). Student’s *t* test was used to compare mean values between control and tested groups, whereas differences between mean values of multiple groups were analyzed by one-way ANOVA with multiple comparison tests, as indicated in the figure legends. Significance is indicated in the figure legends.

## Data availability

All data are available in the main text or the supporting information.

## Supporting information

This article contains supporting information.

## Conflict of interest

The authors declare that they have no conflicts of interest with the contents of this article.

## References

[1] Stival C., Puga Molina Ldel C., Paudel B., Buffone M.G., Visconti P.E., Krapf D. (2016). Sperm capacitation and acrosome reaction in mammalian sperm. Adv. Anat. Embryol. Cell Biol..

[2] Santi C.M., Martínez-López P., de la Vega-Beltrán J.L., Butler A., Alisio A., Darszon A. (2010). The SLO3 sperm-specific potassium channel plays a vital role in male fertility. FEBS Lett..

[3] Matamoros-Volante A., Trevino C.L. (2020). Capacitation-associated alkalization in human sperm is differentially controlled at the subcellular level. J. Cell Sci..

[4] Baro Graf C., Ritagliati C., Torres-Monserrat V., Stival C., Carizza C., Buffone M.G. (2019). Membrane potential assessment by fluorimetry as a predictor tool of human sperm fertilizing capacity. Front. Cell Dev. Biol..

[5] Ren D., Navarro B., Perez G., Jackson A.C., Hsu S., Shi Q. (2001). A sperm ion channel required for sperm motility and male fertility. Nature.

[6] Wang D., Hu J., Bobulescu I.A., Quill T.A., McLeroy P., Moe O.W. (2007). A sperm-specific Na+/H+ exchanger (sNHE) is critical for expression and in vivo bicarbonate regulation of the soluble adenylyl cyclase (sAC). Proc. Natl. Acad. Sci. U. S. A..

[7] Nishigaki T., José O., González-Cota A.L., Romero F., Treviño C.L., Darszon A. (2014). Intracellular pH in sperm physiology. Biochem. Biophysical Res. Commun..

[8] Gardner C.C., James P.F. (2023). Na+/H+ exchangers (NHEs) in mammalian sperm: essential contributors to male fertility. Int. J. Mol. Sci..

[9] Gardner C.C., James P.F. (2023). The SLC9C2 gene product (Na+/H+ exchanger isoform 11; NHE11) is a testis-specific protein localized to the head of mature mammalian sperm. Int. J. Mol. Sci..

[10] Woo A.L., James P.F., Lingrel J.B. (2002). Roles of the Na,K-ATPase α4 isoform and the Na+/H+ exchanger in sperm motility. Mol. Reprod. Dev..

[11] Bell S.M., Schreiner C.M., Schultheis P.J., Miller M.L., Evans R.L., Vorhees C.V. (1999). Targeted disruption of the murine Nhe1 locus induces ataxia, growth retardation, and seizures. Am. J. Physiol..

[12] Chen S.R., Chen M., Deng S.L., Hao X.X., Wang X.X., Liu Y.X. (2016). Sodium–hydrogen exchanger NHA1 and NHA2 control sperm motility and male fertility. Cell Death Dis..

[13] Balbach M., Hamzeh H., Jikeli J.F., Brenker C., Schiffer C., Hansen J.N. (2020). Molecular mechanism underlying the action of zona-pellucida glycoproteins on mouse sperm. Front. Cell Dev Biol..

[14] Windler F., Bönigk W., Körschen H.G., Grahn E., Strünker T., Seifert R. (2018). The solute carrier SLC9C1 is a Na+/H+-exchanger gated by an S4-type voltage-sensor and cyclic-nucleotide binding. Nat. Commun..

[15] Lishko P.V., Kirichok Y., Ren D., Navarro B., Chung J.J., Clapham D.E. (2012). The control of male fertility by spermatozoan ion channels. Annu. Rev. Physiol..

[16] Baro Graf C., Ritagliati C., Stival C., Luque G.M., Gentile I., Buffone M.G. (2020). Everything you ever wanted to know about PKA regulation and its involvement in mammalian sperm capacitation. Mol. Cell Endocrinol..

[17] Escoffier J., Krapf D., Navarrete F., Darszon A., Visconti P.E. (2012). Flow cytometry analysis reveals a decrease in intracellular sodium during sperm capacitation. J. Cell Sci..

[18] Weeks K.L., Ke P., Zhang J., Zhang X., Chen X. (2020). Protein kinase inhibitor peptide as a tool to specifically inhibit protein kinase A. Front. Physiol..

[19] Krapf D., Arcelay E., Wertheimer E.V., Sanjay A., Pilder S.H., Salicioni A.M. (2010). Inhibition of Ser/Thr phosphatases induces capacitation-associated signaling in the presence of Src kinase inhibitors. J. Biol. Chem..

[20] Wrighton D.C., Muench S.P., Lippiat J.D. (2015). Mechanism of inhibition of mouse Slo3 (KCa5.1) potassium channels by quinine, quinidine and barium. Br. J. Pharmacol..

[21] Schaap P., van Ments-Cohen M., Soede R.D., Brandt R., Firtel R.A., Dostmann W. (1993). Cell-permeable non-hydrolyzable cAMP derivatives as tools for analysis of signaling pathways controlling gene regulation in Dictyostelium. J. Biol. Chem..

[22] Wertheimer E., Krapf D., de la Vega-Beltran J.L., Sánchez-Cárdenas C., Navarrete F., Haddad D. (2013). Compartmentalization of distinct cAMP signaling pathways in mammalian sperm. J. Biol. Chem..

[23] Balbach M., Ghanem L., Rossetti T., Kaur N., Ritagliati C., Ferreira J. (2021). Soluble adenylyl cyclase inhibition prevents human sperm functions essential for fertilization. Mol. Hum. Reprod..

[24] Lucchesi O., Ruete M.C., Bustos M.A., Quevedo M.F., Tomes C.N. (2016). The signaling module cAMP/Epac/Rap1/PLCε/IP3 mobilizes acrosomal calcium during sperm exocytosis. Biochim. Biophys. Acta.

[25] Wang D., King S.M., Quill T.A., Doolittle L.K., Garbers D.L. (2003). A new sperm-specific Na+/H+ exchanger required for sperm motility and fertility. Nat. Cell Biol..

[26] Chávez J.C., Ferreira J.J., Butler A., De La Vega Beltrán J.L., Treviño C.L., Darszon A. (2014). SLO3 K+ channels control calcium entry through CATSPER channels in sperm. J. Biol. Chem..

[27] Graf C.B., Ritagliati C., Stival C., Balestrini P.A., Buffone M.G., Krapf D. (2019). Determination of a robust assay for human sperm membrane potential analysis. Front. Cell Dev Biol..

[28] Hernández-Garduño S., Chávez J.C., Matamoros-Volante A., Sánchez-Guevara Y., Torres P., Treviño C.L. (2022). Hyperpolarization induces cytosolic alkalization of mouse sperm flagellum probably through sperm Na+/H+ exchanger. Reproduction.

[29] Masereel B., Pochet L., Laeckmann D. (2003). An overview of inhibitors of Na+/H+ exchanger. Eur. J. Med. Chem..

[30] Kang H., Liu M., Zhang W., Huang R.Z., Zhao N., Chen C. (2021). Na+/H+ exchangers involve in regulating the ph-sensitive ion channels in mouse sperm. Int. J. Mol. Sci..

[31] Muzzachi S., Guerra L., Martino N.A., Favia M., Punzi G., Silvestre F. (2018). Effect of cariporide on ram sperm pH regulation and motility: possible role of NHE1. Reproduction.

[32] Nørholm A.-B., Hendus-Altenburger R., Bjerre G., Kjaergaard M., Pedersen S.F., Kragelund B.B. (2011). The intracellular distal tail of the Na+/H+ exchanger NHE1 is intrinsically disordered: implications for NHE1 trafficking. Biochemistry.

[33] Köster S., Pavkov-Keller T., Kühlbrandt W., Yildiz O. (2011). Structure of human Na +/H + exchanger NHE1 regulatory region in complex with calmodulin and Ca 2+. J. Biol. Chem..

[34] Wakabayashi S., Bertrand B., Ikeda T., Pouysségur J., Shigekawa M. (1994). Mutation of calmodulin-binding site renders the Na+/H+ exchanger (NHE1) highly H+-sensitive and Ca2+ regulation-defective. J. Biol. Chem..

[35] Navarrete F.A., García-Vázquez F.A., Alvau A., Escoffier J., Krapf D., Sánchez-Cárdenas C. (2015). Biphasic role of calcium in mouse sperm capacitation signaling pathways. J. Cell Physiol..

[36] Zeng X.H., Yang C., Kim S.T., Lingle C.J., Xia X.M. (2011). Deletion of the Slo3 gene abolishes alkalizationactivated K+ current in mouse spermatozoa. Proc. Natl. Acad. Sci. U. S. A..

[37] Escoffier J., Navarrete F., Haddad D., Santi C.M., Darszon A., Visconti P.E. (2015). Flow cytometry analysis reveals that only a subpopulation of mouse sperm undergoes hyperpolarization during capacitation. Biol. Reprod..

[38] Limbutara K., Kelleher A., Yang C.R., Raghuram V., Knepper M.A. (2019). Phosphorylation changes in response to kinase inhibitor H89 in PKA-null cells. Sci. Rep..

[39] Chen Y., Sabatini B.L. (2021). The kinase specificity of protein kinase inhibitor peptide. Front. Pharmacol..

[40] Navarro B., Kirichok Y., Clapham D.E. (2007). KSper, a pH-sensitive K+ current that controls sperm membrane potential. Proc. Natl. Acad. Sci. U. S. A..

[41] Lyon M.D., Ferreira J.J., Li P., Bhagwat S., Butler A., Anderson K. (2023). SLO3: a conserved regulator of sperm membrane potential. Int. J. Mol. Sci..

[42] Dolan J., Walshe K., Alsbury S., Hokamp K., O'Keeffe S., Okafuji T. (2007). The extracellular leucine-rich repeat superfamily; a comparative survey and analysis of evolutionary relationships and expression patterns. BMC Genomics.

[43] Quill T.A., Wang D., Garbers D.L. (2006). Insights into sperm cell motility signaling through sNHE and the CatSpers. Mol. Cell Endocrinol..

[44] Cavarocchi E., Whitfield M., Chargui A., Stouvenel L., Lorès P., Coutton C. (2021). The sodium/proton exchanger SLC9C1 (sNHE) is essential for human sperm motility and fertility. Clin. Genet..

[45] Scholz W., Albus U., Counillon L., Gögelein H., Lang H.J., Linz W. (1995). Protective effects of HOE642, a selective sodium-hydrogen exchange subtype 1 inhibitor, on cardiac ischaemia and reperfusion. Cardiovasc. Res..

[46] Grahn E., Kaufmann S.V., Askarova M., Ninov M., Welp L.M., Berger T.K. (2023). Control of intracellular pH and bicarbonate by CO2 diffusion into human sperm. Nat. Commun..

[47] Ritagliati C., Baro Graf C., Stival C., Krapf D. (2018). Regulation mechanisms and implications of sperm membrane hyperpolarization. Mech. Dev..

[48] Ritagliati C., Luque G.M., Stival C., Baro Graf C., Buffone M.G., Krapf D. (2018). Lysine acetylation modulates mouse sperm capacitation. Sci. Rep..

[49] Luque G.M., Xu X., Romarowski A., Gervasi M.G., Orta G., De la Vega-Beltrán J.L. (2021). Cdc42 localized in the CatSper signaling complex regulates cAMP-dependent pathways in mouse sperm. FASEB J..

